# HTLV-1 bZIP factor-mediated dysfunction of regulatory T cells in vivo

**DOI:** 10.1186/1742-4690-8-S1-A111

**Published:** 2011-06-06

**Authors:** Paola Miyazato, Yorifumi Satou, Tomoyuki Yamaguchi, Shimon Sakaguchi, Kouichi Ohshima, Masao Matsuoka

**Affiliations:** 1 Laboratory of Virus Control, Institute for Virus Research, Kyoto University, Kyoto, Japan; 2 Department of Experimental Pathology, Institute for Frontier Medical Sciences, Kyoto University, Kyoto, Japan; 3 Department of Pathology, Kurume University School of Medicine, Kurume, Fukuoka, Japan

## 

Among the accessory proteins important for the biology of the Human T cell leukemia virus type 1 (HTLV-1) infection, HTLV-1 bZIP factor (HBZ) was previously reported to be a key player in the proliferation of infected cells. We recently showed that HBZ affects the regulatory T cell (Treg) population by impairing their suppressive function, despite the fact that it increases the expression of the Treg master molecule, the transcription factor Foxp3.

In the present study, we further characterize the effect of HBZ on this particular population in an in vivo model of colitis. It has been known that inoculation of naïve T cells induces T cell-mediated colitis in the lymphocyte-deficient Rag-/- mice, and that co-injection of Tregs prevents the disease. Rag-/- mice were inoculated with naïve T cells in the presence of Tregs from wild type (WT) or HBZ-transgenic (Tg) mice. Histo-pathological analysis of colon sections showed more intensive infiltration of lymphocytes in the group of mice receiving HBZ-Tg Tregs, compared to the one receiving WT-Tregs. In order to better understand the basis of these results, we transduced mouse naïve T cells with retroviral vectors expressing HBZ and/or Foxp3, and analyzed the expression of Treg-associated molecules. We found that HBZ affected the transcriptional activity of Foxp3, resulting in the abnormal expression of these molecules.

Our results suggest that HBZ leads to the malfunction of Tregs, resulting in inflammatory symptoms in HTLV-1-associated diseases.

